# Efficacy and Safety of Keratosept Eye Drops in Patients with Punctate Keratitis: Clinical and Microbiological Evaluation on 50 Eyes

**DOI:** 10.3390/microorganisms12112277

**Published:** 2024-11-09

**Authors:** Mario Troisi, Ciro Costagliola, Michele Rinaldi, Diego Strianese, Elisabetta Chiariello Vecchio, Salvatore Troisi

**Affiliations:** 1 Eye Clinic, Department of Neurosciences, Reproductive and Odontostomatological Sciences, University of Naples Federico II, 80131 Naples, Italy; ciro.costagliola@unina.it (C.C.); michele.rinaldi2@unina.it (M.R.); diego.strianese@unina.it (D.S.); 2 Ophthalmologic Unit, Salerno Hospital University, 84100 Salerno, Italy; elisacv@gmail.com

**Keywords:** Keratosept, antiseptic eye drops, punctate keratitis, hexamethidine disethionate, polyhexanichlorohydrate, edetate disodium

## Abstract

We evaluated the efficacy and safety of eye drops with antiseptic and re-epithelizing properties (Keratosept^®^, Bruschettini, Genova, Italy) on 50 eyes affected by punctate keratitis of suspected microbial origin. A biomicroscopic examination, fluorescein dye staining test (Fluotest), tear break-up time test (TBUT), and the ocular surface disease Index (OSDI) questionnaire were used to assess treatment efficacy. Treatment success was defined as a negative Fluotest and an OSDI score <12 on the 15th day of treatment. According to this definition, Keratosept^®^, either alone or in combination with antibiotics, was effective in over 80% of microbial and nonmicrobial forms. Conjunctival swabs were taken from all patients for culture examination with an antibiogram and sensitivity test for Keratosept^®^; 32/43 (74.4%) forms with positive culture for the bacteria were sensitive to Keratosept^®^. A total of 35/38 (92%) eyes treated with this product alone achieved full treatment success without any apparent adverse effects. These results suggest the use of Keratosept^®^ eye drops in punctate keratitis as an alternative or in combination with established antibiotic therapies. Further studies are needed to evaluate its efficacy in different infectious forms and identify other indications for using this product.

## 1. Introduction

The global rise in keratitis cases caused by multidrug-resistant bacteria has become a significant public health concern, complicating treatment outcomes and highlighting the urgent need for alternative therapeutic approaches [1]. The growing increase in bacterial resistance and the low efficacy of antibiotics commonly used in many infectious forms have led the scientific community to focus their interest on products with antiseptic activity, especially in fields where topical therapies can be performed, such as dermatology, ophthalmology, and stomatology [2,3,4].

In the ophthalmological field, various antiseptics have recently been introduced into clinical practice, generally based on biguanides, chlorhexidine, ethylenediamine-tetraacetate, iodopovidone, or ozonated oil micelles [5,6,7,8,9,10]. Some natural products containing bioflavonoids extracted from citrus fruits have also been proposed as antimicrobials [11,12,13]. Antiseptics could be combined with standard treatments (e.g., antibiotics, antivirals, or antifungals) or with innovative therapeutic protocols, like immune-modulatory therapies, such as nanoparticle-based approaches, which have demonstrated promising effects by altering immune cell behavior to promote healing and reduce inflammation [14].

The purpose of this study was to evaluate the efficacy and safety of Keratosept^®^ (Bruschettini, Genova, Italy) in patients with punctate keratoconjunctivitis of suspected microbial origin. Keratosept^®^ is an antiseptic based on polyvinyl alcohol 1.25%, dexpanthenol, hexamethidine disethionate 0.05%, polyhexaneichlorohydrate 0.0001%, methylsulfonylmethane, and edetate disodium. In particular, we intended to evaluate whether this formulation has anti-infectious and epitheliotropic properties. Keratosept^®^’s mechanism involves the combined action of dexpanthenol, which supports corneal epithelial repair by acting as a precursor to coenzyme A, facilitating tissue repair, and antiseptic components like polyhexanide and hexamethidine disethionate, which disrupt bacterial cell membranes and lead to microbial death [15]. This dual mechanism promotes microbial inhibition and fosters epithelial healing by reducing inflammation and maintaining an ideal environment for healing [16]. In a recent study, this formulation showed good antimicrobial activity on all tested microbial strains, excluding *Pseudomonas aeruginosa*; the ophthalmic solution and its components were safe and non-toxic for corneal and conjunctival epithelial cells for 5 and 10 min at the concentrations analyzed [16].

The present study was undertaken to verify the possible role of this ophthalmic product in the treatment of conjunctival inflammations associated with superficial punctate keratitis (SPK). SPK is a significant cause of ocular morbidity worldwide, which can arise from infectious and non-infectious causes [17,18,19,20]. Non-infectious forms can be related to a variety of systemic or local causes, predominantly of autoimmune, toxic–allergic, or dry eye disease origin [21,22,23]. Fortunately, most cases are successfully managed with medical therapy. However, if treatment failure occurs, they can lead to an ulcerative form with possible devastating consequences, such as vision or eye loss [24]. Nonulcerative corneal inflammation may affect either the epithelial layer, the stroma, or both, impacting disease progression and treatment. This is supported by recent studies on corneal layer involvement in punctate keratitis, which can aid in targeting specific treatments [25].

In this work, patients with exclusively epithelial corneal involvement, associated with signs of ocular surface inflammation of suspected bacterial origin, were enrolled.

This investigation is based on the evaluation of clinical symptoms and signs and on the results of bacterial culture. Side effects, if any, are also evaluated and recorded during this study.

## 2. Materials and Methods

**Figure 1:**
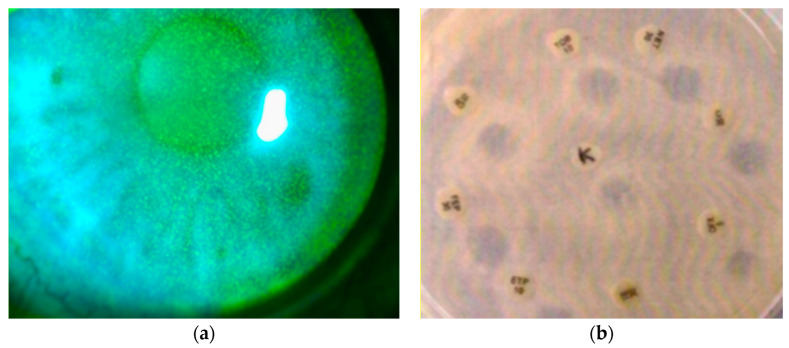
(**a**) Representative image of keratopathy punctata; (**b**) in vitro sensitivity test for antibiotics and Keratosept^®^.

Fifty eyes of 34 consecutive patients with superficial punctate keratoconjunctivitis of a suspected microbial nature were evaluated (Figure 1a).

Inclusion criteria were age > 18 years, the absence of topical ocular therapies in the past seven days, a positive corneal Fluotest in grades I, II, or III of the Oxford scheme, and the suspicion of infection due to discharge or other signs or symptoms.

Exclusion criteria included corneal ulcerations > 1 mm, moderate or severe dry eye, glaucoma, immunosuppression, previous ophthalmic herpes, lagophthalmos, trichiasis, and other local pathological conditions that could induce ocular surface epitheliopathy.

At patient recruitment (baseline), biomicroscopic examination, the fluorescein dye staining test (Fluotest), the tear break-up time test (TBUT), and the ocular surface disease Index (OSDI) questionnaire were performed. Keratosept^®^ eye drops were prescribed at baseline with a posology of one drop every 6 h while awaiting laboratory results.

The brushing of the upper and lower tarsal conjunctiva epithelium was also carried out at baseline for culture tests for bacteria and fungi and sensitivity tests for the commonly used ophthalmological antibiotics and Keratosept^®^. The Kirby–Bauer diffusion method was used to evaluate the effectiveness of this product on isolated germs [26]. A zone of inhibition >10 mm four hours after culture was considered an indication of positive eye drop sensitivity (Figure 1b).

Therapeutic re-evaluation was performed five days after baseline, on the basis of the results of culture tests and an antibiogram. For microorganisms sensitive to Keratosept^®^ or in the presence of negative cultures, treatment with Keratosept^®^ eye drops alone continued through day 15. In the case of germs not sensitive to Keratosept^®^, targeted antibiotic eye drops were added to Keratosept^®^.

Clinical re-evaluation was conducted at 10 and 15 days, assessing the evolution of clinical signs (biomicroscopic examination, TBUT, Fluotest) and symptoms (OSDI).

Patients with a negative Fluotest and an OSDI score < 12 on the 15th day were considered cured. Those with persistent epithelial defects or an OSDI score ≥ 12 underwent microbiological re-evaluation after a 5-day treatment washout (day 20 after baseline). The samples were cultured for aerobic and anaerobic bacteria and fungi using routine microbiological techniques.

Non-parametric tests (Mann–Whitney, IBM SPSSStatistics V.29, Chicago, IL, USA) were applied to compare the within-group evolution (versus the previous time point) of the TBUT, Fluotest, and OSDI questionnaire.

## 3. Results

A total of 43 out of 50 conjunctival curettage samples submitted to culture (86%) showed bacterial development (28 Gram +, 10 Gram −, and 4 mixed flora); 1 case of *Candida albicans* infection was also reported (Table 1). Six cases had negative microbial cultures. A total of 32 of the 43 positive bacterial cultures (74%) had a Keratosept^®^ inhibition zone > 10 mm on the antibiogram and were defined as Keratosept^®^-sensitive (Figure 1b, Table 1).

**Figure 2:**
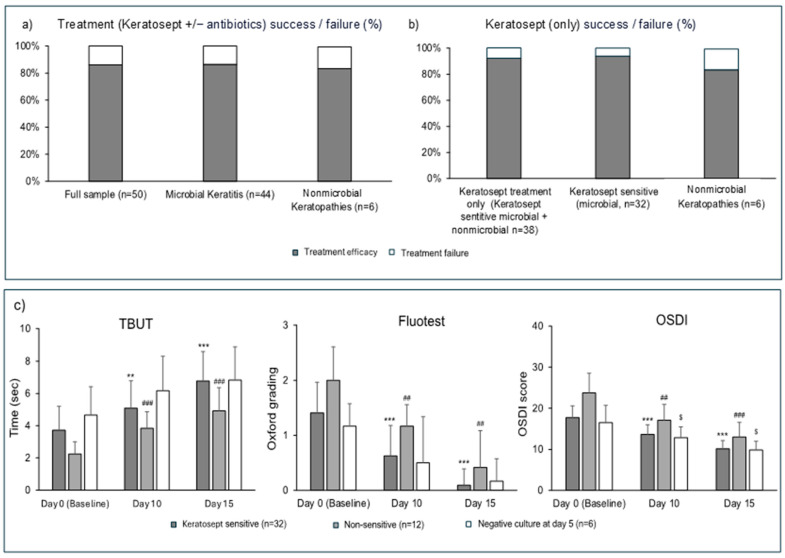
(**a**) Treatment success (either Keratosept^®^ alone or in combination with targeted antibiotics) in the overall sample (n = 50) and by microbial (n = 44) and nonmicrobial (n = 6) keratopathies. (**b**) Treatment success proportions in cases treated with Keratosept only and by Keratosept^®^-sensitive and nonmicrobial keratopathies. (**c**) A longitudinal evolution of the fluorescein dye staining test (Fluotest), the tear break-up time test (TBUT), and the ocular surface disease Index (OSDI) questionnaire by subgroups. *** *p* < 0.001 and ** *p* < 0.01 vs. the previous time point within the “Keratosept^®^-sensitive” group. ^###^ *p* < 0.001 and ^##^ *p* < 0.01 vs. the previous time point within the “Non-sensitive” group; ^$^ *p* < 0.05 vs. the previous time point within the “Negative culture at day 5” group; Mann–Whitney non-parametric test.

Treatment success, defined as a negative Fluotest and an OSDI <12 on day 15, occurred in 43/50 eyes overall (86%, Figure 2a).

Keratosept^®^-sensitive cases (n = 32) and those with negative cultures (n = 6) were treated with Keratosept^®^ only through day 15, achieving therapeutic success in 35/38 eyes (92%, Figure 2b). Regarding Keratosept^®^-sensitive eyes, clinical recovery was recorded for 30/32 of the cases (93%). In the remaining two Keratosept^®^-sensitive cases, there was grade I corneal epitheliopathy (Oxford scheme) on day 15. A new microbiological examination was performed after five days of Keratosept^®^ washout (on day 20), and the results showed a negative culture. A severe dry eye diagnosis was made in these two eyes.

Keratosept^®^-sensitive eyes showed a significant improvement in TBUT, Fluotest, and OSDI through days 10 to 15 (Figure 2c). In the case of nonmicrobial keratopathies, there was a numerical improvement in the TBUT and Fluotest and a significant reduction in symptoms through days 10 to 15.

Combination treatment (Keratosept^®^ + antibiotics) for microbial keratitis in non-Keratosept^®^-sensitive eyes was successful in 8/12 of the cases (67%). Improvements in the TBUT, Fluotest, and OSDI were significant through day 15. From the remaining four cases where the combination therapy failed, one eye was positive for methicillin-resistant *St. aureus* (MRSA), and another case showed the persistence of *Candida albicans* infection, despite the association of eye drops with Fluconazole. The punctate keratopathy improved in the remaining two eyes, although it persisted due to a previously unrecognized dry eye disease.

Keratosept^®^ was well tolerated by all patients without apparent side effects. Notably, symptom improvement was also reported in subjects with negative cultures. The average reduction in the OSDI score in subjects with negative cultures was 5.71 points. In the 43 patients with positive cultures, a mean improvement in the OSDI score of 8.6 points from baseline was observed on day 15 of therapy.

## 4. Discussion

Antibiotic-resistant bacteria are continuously emerging due to the widespread and sometimes indiscriminate use of antibiotics in medicine [27]. Resistance to antibiotics is a natural expression of bacterial evolution and genetics; the more a particular antibiotic is used, the greater the chance of developing a resistance problem [28]. Over time, multidrug resistance can be acquired through the gradual accumulation of mutations or through the acquisition of resistance genes from other bacteria [29]. In many cases, antiseptics can be a valuable adjuvant and often also an alternative to antibiotics for the prevention and treatment of infections, reducing the excessive use of antibiotics [30]. Compared to antibiotics commonly used in ophthalmology, Keratosept^®^ demonstrates a favorable safety profile. Its combination with non-toxic hydrating agents like polyvinyl alcohol helps minimize ocular surface cytotoxicity often observed with aminoglycosides and fluoroquinolones. Keratosept^®^’s broad-spectrum antiseptic action reduces hypersensitivity risks and irritation typically caused by antibiotic eye drops [16,31]. Moreover, the broad-spectrum antibacterial properties of Keratosept, especially due to polyhexanide, minimize the risk of developing microbial resistance [16]. Polyhexanide belongs to the same pharmaceutical family as chlorhexidine and is active against a wide range of bacteria, fungi, and parasites. Its activity is thought to be due to its rapid attraction towards the negatively charged phospholipids at the bacterial cell surface, impairing membrane function by causing potassium ions to be lost and intracellular components to precipitate [32].

Unlike antibiotics, which target specific bacterial pathways, antiseptics like Keratosept act through multiple non-specific mechanisms, reducing the chance of resistance. Antiseptics have a non-selective mechanism of action, which generally prevents the development of resistance [33]. For this reason, there are less frequent reports suggesting that the development of antiseptic-resistant microorganisms may increase with the widespread use of these agents [34]. Furthermore, antiseptics are also effective on some nonbacterial microorganisms [30].

In comparison with other antiseptics and antibiotics, Keratosept demonstrates notable advantages, especially in nonmicrobial keratitis and in cases where antibiotic resistance limits traditional treatment options. While chlorhexidine and iodopovidone can irritate the ocular surface, Keratosept’s dexpanthenol content reduces such side effects while promoting epithelial repair. Chlorhexidine has been shown to be effective in treating Acanthamoeba keratitis but may cause toxicity with prolonged use [35], while povidone-iodine is a broad-spectrum antiseptic often limited by ocular surface irritation [36]. In contrast, Keratosept’s formulation with dexpanthenol helps mitigate these side effects while promoting epithelial healing. Studies also show that moxifloxacin and tobramycin are effective for bacterial keratitis but face increasing resistance, which limits their efficacy [37]. In nonmicrobial keratitis, antibiotics offer limited benefits and can worsen epithelial damage, making Keratosept a safer and more effective alternative.

Superficial punctate keratopathy represents a therapeutic problem, as it can be a consequence of various etiopathogenetic mechanisms. Epithelial defects of infectious origin can be caused by pathogenic microorganisms of various kinds, such as bacteria, viruses, fungi, and protozoa [38]. In these cases, the use of an antiseptic instead of an antibiotic often allows for the attack of even nonbacterial microorganisms, reaching a broader spectrum of action. Furthermore, non-infectious forms often tend to worsen following the local administration of antibiotics, which can have cytotoxic effects on the ocular surface. Existing recommendations on local antimicrobial agents in punctate keratopathies do not contain references to specific products and do not explain their main characteristics. The role of antiseptics and topical formulations of antibiotics in the treatment of these forms is not fully defined, which gives the impression of identical indications for their use. Topical formulations, on the other hand, differ significantly in their chemical structure and mechanism of action, as evidenced by scanning electron microscopy and clinical studies [39]. Therapeutic recommendations for punctate keratopathies need to be tailored to the specific conditions of the disease, as many antiseptics and antibiotics have a negative impact on the course of wound healing when applied topically [40].

In the present study, Keratosept^®^ eye drops, without the addition of other treatments, were effective in 35 of the 50 eyes affected by punctate keratitis (70%). The clinical result obtained is associated with a high percentage of positivity (72%) of the inhibition halo (>10 mm) determined for this product on the culture plates. These values are similar to those reported by traditional antibiotics (efficacy of about 70% on bacterial strains isolated by conjunctival brushing) [17]. Moreover, all patients considered sensitive to Keratosept^®^ showed clinical recovery or negative results in a subsequent microbiological examination in the case of the persistence of epithelial defects. The high concordance between sensitivity to Keratosept^®^ in the culture plates and the clinical and microbiological result of the treatment confirms the validity of the evaluation method adopted.

The main advantage of using antiseptics over antibiotics is the lower tendency to cause bacterial resistance. It is also worth noting that the presence of polyvinyl alcohol in the Keratosept^®^ formulation improves the hydration of the ocular surface and favors a dilution of any bacterial load. Moreover, the presence of dexpanthenol exerts a cytoprotective and repairing effect on the structures of the ocular surface [13]. These characteristics probably favored complete re-epithelialization in seven out of eight eyes with punctate keratopathy and negative microbiological tests after 15 days of therapy with Keratosept^®^. Notably, all patients in this subgroup showed a significant improvement in their symptoms, denoted by a mean OSDI test score of 5.71 points. This finding is particularly important in the therapy of punctate keratopathy, where sometimes, the use of antibiotics leads to cytotoxicity to the ocular surface system. The results obtained with the treatment with Keratosept^®^ appear even more interesting if we consider that four of the seven eyes infected with germs not sensitive to Keratosept^®^ did not heal despite the use of specific antibiotics for ten days. We also believe, on the basis of literature data, that in the product used, the association of several compounds with antiseptic activity can minimize the risk of developing resistance. Furthermore, the reduced concentration of each component improves its tolerability.

These evaluations make the use of Keratosept^®^ very interesting in cases of epithelial defects with or without bacterial infection. Many cases of nonmicrobial epithelial defects present high risks of secondary infection, for example, in the presence of allergic conjunctivitis, dry eye, viral forms, the use of contact lenses, trauma, morphological and functional alterations in the eyelids, and floppy eyelid syndrome [41,42,43]. In these conditions, it is very advantageous to administer a lubricating and epitheliotropic product that also protects against infectious complications.

Some authors have published works showing a valid in vitro antimicrobial activity of Keratosept^®^ on Staphylococci, Streptococci, other bacterial species, and even on Candida [15,16,31]. For instance, Modugno et al. reported a clinical evaluation of the antimicrobial activity and ocular tolerability of this product in patients undergoing cataract surgery [31]. However, the present study is the first carried out on the in vivo efficacy of Keratosept^®^ on eyes affected by superficial punctate keratopathy, an insidious pathologic condition of various etiologies and not always easy to identify [44,45]. In infectious forms, the correlation between in vitro antimicrobial activity and clinical data confirms the validity of the results. The data collected on non-infectious forms also show a significant improvement in the TBUT, Fluotest, and OSDI score.

The authors are aware of the limitations of this study, mainly due to the small sample size, the lack of a control group receiving either a placebo or a standard treatment (e.g., antibiotics) or other topical antiseptics for comparison, and the blinded condition. Another limitation of the current study is the absence of long-term follow-up, which could help in observing any delayed adverse reactions or recurrence of keratitis. A minimum follow-up period of 6 months would allow for a comprehensive evaluation of the persistence of epithelial healing, any signs of chronic inflammation, and the potential for late-onset adverse events, particularly in the case of chronic keratitis management. Furthermore, good compliance is assumed from the absence of reported adverse effects and the improvement in the OSDI questionnaire score, which occurred in all patients. Nevertheless, no specific tools were used, such as the conjunctival hyperemia scale, to objectively evaluate the tolerability of this ophthalmic solution. Compatible with these limitations, the high efficacy demonstrated against pathogenic bacteria also suggests its indication as surgical prophylaxis, as already proposed by other authors, especially where it is necessary to repeat these preventive procedures several times a year, in the case of intravitreal injections of anti-VEGF [46,47].

## 5. Conclusions

The high clinical and microbiological efficacy shown by the product under study confirms its indication in cases of punctate keratitis of infectious or other origin. The absence of side effects in all treated eyes and the re-epithelialization action demonstrated even in cases not associated with infection confirm the efficacy and safety of the product.

The obtained results suggest other indications for this medical device, both for preventive and therapeutic purposes. Further studies are needed to confirm the efficacy of further product indications.

## Figures and Tables

**Table 1:** A summary of the conjunctival swab results.

Swab Results	N (Percentage)
Gram +	28 (56%)
Gram −	10 (20%)
Bacteria mix	4 (8%)
*Candida albicans*	1 (2%)
Negative culture	7 (14%)

## Data Availability

The original contributions presented in the study are included in the article, further inquiries can be directed to the corresponding authors.
